# A qualitative exploration of the patient experience of erosive and non-erosive hand osteoarthritis

**DOI:** 10.1186/s41687-021-00286-1

**Published:** 2021-02-03

**Authors:** Charlotte Panter, Pamela Berry, Deven Chauhan, Sofia Fernandes, Sally Gatsi, Josephine Park, Jane R. Wells, Rob Arbuckle

**Affiliations:** 1 Adelphi Values, Patient-Centered Outcomes, Bollington, Cheshire UK; 2 GSK, Collegeville, PA USA; 3 Present Address: Janssen Global Services LLC, Horsham, PA USA; 4 Value Evidence and Outcomes, Stockley Park West, 1-3 Ironbridge Road, GSK, Uxbridge, Middlesex UB11 1BT UK; 5 GSK, Stevenage, Hertfordshire, UK

**Keywords:** Hand osteoarthritis, Michigan Hand Outcomes Questionnaire, Concept elicitation, Qualitative interviews, Real-time data capture, Patient-experience, Patient-reported outcomes

## Abstract

**Background:**

Many patients with hand osteoarthritis (HOA) experience reduced health-related quality of life. This study sought to better understand the disease and treatment experience of individuals with HOA, explore any differences in experiences between erosive and non-erosive HOA sub-types, and evaluate content validity of the Michigan Hand Outcomes Questionnaire (MHQ) in HOA.

**Methods:**

Thirty subjects from the United States (*n* = 15 erosive HOA; *n* = 15 non-erosive HOA) participated in semi-structured interviews: concept elicitation explored symptoms/impacts important to patients; cognitive interviews assessed understanding and relevance of the MHQ. A sub-sample participated in real-time data capture (RTDC) activities via a smartphone/tablet app over 7 days. Verbatim transcripts were coded using Atlas.ti software and thematically analyzed. Concept saturation and MHQ content validity were evaluated.

**Results:**

Most participants reported experiencing pain, swelling and stiffness, symptoms that most commonly had a direct impact on physical functioning. Substantial impacts on activities of daily living, emotional functioning, sleep and work were also reported. RTDC findings corroborated concept elicitation findings. There were no notable differences between erosive and non-erosive HOA, except nodules were reported more frequently in erosive disease. Most participants used analgesic treatments, but effects were short-lived. Pain was the symptom most frequently reported as most bothersome and important to treat. Concept saturation was achieved. MHQ items and instructions were well understood and relevant to most participants; stiffness and swelling were reported as important symptoms not included in the MHQ.

**Conclusions:**

This study characterizes key symptoms of HOA which are burdensome for patients and not well controlled by current therapies, highlighting an unmet treatment need. Although the study is limited by a small sample size that may not be representative of the broader erosive and non-erosive HOA population, concept saturation was achieved, and our findings suggest that disease experience is similar for patients with erosive and non-erosive HOA. Evaluation of stiffness and swelling items in conjunction with the MHQ may enhance relevance and improve measurement precision to assess important domains of HQRoL in an HOA population.

**Supplementary Information:**

The online version contains supplementary material available at 10.1186/s41687-021-00286-1.

## Background

Hand osteoarthritis (HOA) is among the most common forms of OA, with an age-standardized (40–84 years) prevalence of approximately 40% in the US [1, 2]. HOA is localized to the hand/wrist and is characterized by joint pain/aching, stiffness and swelling, decreased range of motion, loss of strength, and impaired grip [1, 3, 4]. As such, patients with HOA can experience reduced health-related quality of life (HRQoL) including impairments in physical functioning and mental/emotional well-being [5–7].

Erosive HOA is characterized by rapid onset and more inflammatory signs including stiffness, swelling and erythema, more synovitis, and a more aggressive disease course than non-erosive HOA [8–11]. However, there are limited data comparing the patient experience of erosive versus non-erosive forms of HOA.

Qualitative, concept elicitation interviews are an established means of gaining in-depth insight into the patient experience of disease and its treatment, and are an important method for designing patient-centric clinical trial measurement strategies [12, 13]. The increased availability of personal technology in recent years has allowed for the development of novel exploratory approaches to complement traditional interviews. Smartphone/tablet apps offer the possibility to collect patient experience data in ‘real-time’, capturing disease symptoms and impacts as they are experienced.

Important concepts identified from patient insights can inform the selection of suitable patient-reported outcome (PRO) instruments for use in clinical trials, observational studies, and clinical practice. Indeed, guidelines have been developed for both the development of PRO measures for clinical studies, and the selection and implementation of them in practice [12, 14]. While the Australian Canadian Osteoarthritis Hand Index (AUSCAN) is widely used and has been developed for use in HOA [15, 16], as indicated in a study of its use in patients with rheumatoid arthritis, it is a measure of pain, stiffness and function, and does not measure the emotional and behavioral consequences of pain [17]. The Michigan Hand Outcomes Questionnaire (MHQ) is a PRO instrument designed to assess HRQoL domains relevant to individuals with a variety of hand and upper extremity injuries and conditions [18, 19]. The MHQ has been shown to have good responsiveness and reliability across a wide variation of conditions including carpal tunnel syndrome [20], rheumatoid arthritis [21] and distal radius fracture [22], and has previously demonstrated adequate psychometric properties, including internal consistency, test-retest reliability and construct validity, for its use in evaluation of hand function in patients with OA [23]. It assesses hand outcomes across six domains: overall hand function, activities of daily living (ADL), work performance, pain, aesthetics and satisfaction with hand function [19].

Overall, as well as assessing disability, the MHQ allows the assessment of aspects including patient satisfaction, aesthetics and behavioral and emotional effects of pain, which are also relevant to understanding the patient experience of disease [17]. In comparison, the AUSCAN only assesses outcomes across three domains: pain, stiffness and function [23]. The MHQ may also have slightly higher sensitivity to detect change compared with the AUSCAN [10, 23, 24]. Furthermore, features of the MHQ, namely the ability to assess left and right hand functioning separately, make it a valuable tool when evaluating unilateral interventions [17] and present potential opportunities for more precise measurement of outcomes in HOA. Based on assessing a great number of potentially relevant concepts to HOA and potentially higher sensitivity, the MHQ rather than the AUSCAN was selected for evaluation in this study.

For any PRO instrument, content validity should be established in the target population to ensure it assesses relevant concepts, is adequately comprehensive and is consistently interpreted as intended [12, 25, 26]. Face validity assessment of the MHQ revealed that the instrument focuses on many concepts typically relevant to HOA; however, content validity of the MHQ in an HOA population has not previously been evaluated.

This study aimed to better understand the disease and treatment experience of patients with erosive and non-erosive HOA, and to evaluate the suitability of the MHQ for assessing symptoms and impacts of HOA in a clinical trial setting*.*

## Methods

### Objectives

The objectives of this study were to: investigate through patient interviews the experience of erosive and non-erosive HOA symptoms and impacts, and patient satisfaction with current treatments to develop a conceptual model outlining this experience; explore any differences in the erosive and non-erosive HOA patient experience; capture additional insights from a subset of patients using a smartphone/tablet app; and explore the content validity of the MHQ using cognitive interviews and mapping the concepts assessed in the MHQ to the conceptual model.

### Study design

**Fig. 1:**
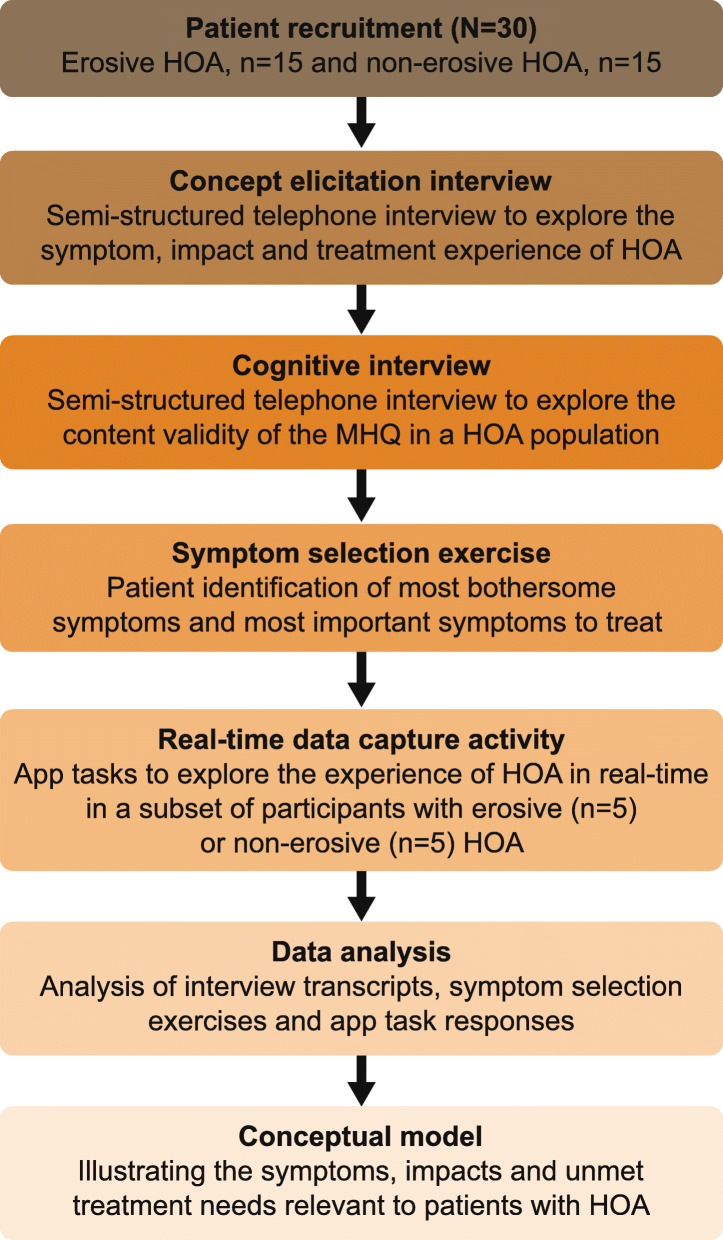
Study methodology. Note: the order of the concept elicitation interview, cognitive interview and symptom selection exercise was not fixed. Most patients completed the concept elicitation and cognitive interviews together, followed by the symptom selection exercise on a different day, but some completed the concept elicitation and cognitive interviews separately, with the symptom selection exercise in between. HOA: Hand osteoarthritis; MHQ: Michigan Hand Outcomes Questionnaire

This study was a non-interventional, cross-sectional qualitative interview study with a prospective, real-time qualitative data capture sub-study, which involved patients with a confirmed diagnosis of HOA in the United States. Participants took part in a concept elicitation interview to identify the symptoms, impacts and treatment experiences important to patients with HOA, and a cognitive interview to assess whether the MHQ is well understood, relevant and captures all concepts important to patients with HOA. Following the interviews, all participants completed a symptom selection exercise and a sub-sample completed real-time data capture (RTDC) activities over 7 days using a bespoke app. The RTDC was exploratory and intended to collect insights in real-time to supplement and verify findings from the concept elicitation interviews (Fig. 1).

### Ethics

The study was approved and overseen by an Independent Review Board in the United States (approval code: ADE1–17-249). Written informed consent was obtained prior to the collection of any data and each participant received $150 upon completion of the two 45-minute telephone interviews as compensation for the time taken to complete the interviews. Those participants who also completed RTDC received an additional payment of $175.

### Participant population

Participants were recruited via physicians/general practitioners and rheumatologists based in three different geographical locations in the United States: Baltimore, MD; Chicago, IL; and New Orleans, LA, between August and September 2017. Physicians identified participants for the study using the inclusion and exclusion criteria. If eligible, and the patient consented to taking part, some further clinical background information was collected using a brief physician-completed case report form (e.g. date of diagnosis and how the diagnosis was made). The diagnosis of (erosive/non-erosive) HOA and study eligibility were confirmed by the physician. Eligible participants (male or female) were 40–80 years of age, met American College of Rheumatology classification of HOA [27] and had self-reported average hand-pain intensity over the past 7 days of ≥4 on a 0 (no pain) to 10 (worst pain imaginable) numerical rating scale. For the erosive sample, participants were required to have X-ray, ultrasound or magnetic resonance imaging evidence of erosive disease in ≥1 distal or proximal interphalangeal joint, and active disease in at least one hand (≥2 swollen/tender distal or proximal interphalangeal joints). For the non-erosive sample, participants were required to have ≥1 finger joint with Kellgren–Lawrence ≥2 [28] by X-ray in the last 12 months, and, in order to ensure a more severe/refractory group that was comparable to the erosive sample, be unwilling/unable to take or have disease inadequately controlled by non-opiate analgesics. Full inclusion and exclusion criteria are provided in the Supplementary Materials. The inclusion/exclusion criteria were developed to be largely reflective of anticipated future clinical trial inclusion and exclusion criteria. A purposive quota sampling approach was taken to ensure the sample was diverse in terms of sex, age, ethnicity, race, highest education level and pain severity.

### Qualitative interviews

The concept elicitation and cognitive interviews were 45 minutes each and conducted by telephone using a semi-structured interview guide; the same study participants completed both interviews. The interviews were either carried out on separate days or the same day, based on patient preference. Interviews were conducted by authors JRW and CP, who are both trained and experienced interviewers from Adelphi Values, with extensive expertise in outcomes research and qualitative methodology. All interviewers participated in a comprehensive briefing and roleplay session prior to conducting any interviews to ensure they were fully familiar with the interview guide and could anticipate and effectively address any difficulties that may arise during the interview. Interviews were audio-recorded and transcribed verbatim for analysis, with all identifiable information redacted. The interview methodology used in this study was in line with published guidance that aims to ensure that qualitative research is rigorous and accurately captures the patient experience [25, 29, 30]. The first 1–2 interviews were reviewed within the project team and the interviewers adapted their use of the semi-structured guide as needed. Transcripts were then thematically analyzed to produce frequency counts, and conceptual models were developed.

The concept elicitation interview started with open-ended, exploratory questioning to facilitate spontaneous and unbiased elicitation of content regarding the patient experience of HOA; for example, “tell me about the arthritis you experience in your hand”. Following this, participants were asked more focused questions designed to probe topics of interest that they may not have mentioned during the interview or concepts/statements that required additional information or clarification; for example, “how long does your stiffness usually last?”. Key topics explored during the interview included signs/symptoms experienced in relation to HOA and impacts on HRQoL (comprising exploration of the following domains: physical functioning, ADL, emotional well-being, social functioning, sleep, work and financial impacts). The findings from the concept elicitation interviews were used to develop a conceptual model of HOA. Following the theory of the Wilson and Cleary model, a conceptual model aims to provide an overview of the disease-related signs/symptoms and how they impact on functional status and various domains of HRQoL, while illustrating the causal relationship among the subjective health constructs [31]. The symptoms and impacts identified were then mapped onto the MHQ to assess concept coverage and suitability for assessing symptoms and impacts of HOA.

For the cognitive interviews, participants were supplied in advanced with a sealed envelope (mailed or given in person) containing the MHQ and advised not to open it until the beginning of the interview to avoid bias. Participants were asked to complete the MHQ using a ‘think aloud’ approach where they were asked to share their reasoning behind each response. Participants were also asked detailed debriefing questions about their understanding of instructions and item wording, the relevance and comprehensiveness of concepts, and the appropriateness of the response options and recall period. For example, “in your own words, what do you understand by this question?”, “is this question relevant to your experience of hand osteoarthritis?”, “is this something you experience?”, and “how far back were you thinking before answering this question?”. These probes were used flexibly, as needed, to ensure that the patient’s understanding of each instruction/item and its relevance was explored, if that was not clear from the patient’s responses to the more open-ended questions.

### Symptom selection exercise

Following the concept elicitation interview, participants were presented with a list of HOA symptoms informed by a brief review of existing qualitative studies and asked to select the most bothersome and most important symptoms to treat.

### Real-time data capture

A subset of participants participated in RTDC as a supplementary and exploratory addition to the qualitative interviews. Patients were recruited on a voluntary basis; the first 10 patients to register interest in both the concept elicitation interviews and RTDC were included in the RTDC subset. These patients downloaded an app to their personal smartphone/tablet and completed a total of 14 tasks over a 7-day period. The app was designed for this study and tailored to collect qualitative and quantitative data relevant to the study objectives. Qualitative tasks were sent at different times throughout each day and were designed to explore symptoms of HOA as they were happening and their impact on different domains of HRQoL (e.g. physical functioning, daily activities) (Supplementary Table S1). Participants responded by submitting images or self-recorded audio or video content via the app. In addition, each morning, participants were also asked to rate the severity of specific symptoms: pain on a 0–10 numerical rating scale, stiffness on a 5-point verbal rating scale (none, mild, moderate, severe and extreme), and swelling on a 5-point verbal rating scale. App responses were sent remotely to a secure database.

### Analysis

Verbatim transcripts of the concept elicitation interviews and RTDC audio responses were subject to thematic analysis [32] using Atlas.ti software (Atlas.ti Scientific Software Development GmbH; Berlin, Germany). Coding was conducted separately by authors JRW and CP. After coding of the first transcript, a meeting was held to to reach a consensus on a provisional code list and coding scheme. Each transcript was assessed and participant comments pertaining to the research questions were highlighted and assigned corresponding concept codes. New codes were added iteratively throughout the analysis. As new codes emerged, previously coded transcripts were reviewed, and the project leader ensured consistency of coding across all transcripts, after which new codes were added if relevant. After analysing each transcript, a list of participant verbatim statements was generated for each coded domain/concept. Further quantitative analysis of this data was not carried out because it was not the purpose of qualitative research and the sample sizes would be too small for meaningful interpretation of results. Visual analysis was conducted on any photo or video data [33].

Concept saturation (i.e. the point at which no new concepts are likely to emerge with continued data collection) is a widely accepted method for determining sample size in qualitative research [34, 35]. Saturation for the concept elicitation interviews was evaluated by dividing transcripts into three equal sets and identifying if any new concepts were spontaneously elicited in the final set of transcripts. This analysis was conducted in the total sample and within erosive and non-erosive sub-samples.

Cognitive interview transcripts were analyzed and a frequency count for each item in the MHQ was provided for understanding, relevance, appropriateness of response options, and adherence to and appropriateness of recall period. Frequency counts were also provided for other select codes of interest, such as the number of participants who suggested a change to an item.

## Results

### Participant sample

Thirty English-speaking participants from the United States with erosive (*n* = 15) or non-erosive (*n* = 15) HOA were interviewed. Participants were recruited from Baltimore (*n* = 5 erosive; *n* = 6 non-erosive), Chicago (*n* = 7 erosive; *n* = 6 non-erosive) and New Orleans (*n* = 3 erosive; *n* = 3 non-erosive). Of these, 10 (*n* = 5 erosive; *n* = 5 non-erosive) completed the RTDC app activity. All participants had received a diagnosis of HOA via X-ray (a requirement for eligibility), and for most a clinical examination was also performed (*n* = 28/30, 93%). Demographics and clinical characteristics are shown in Table 1. The mean age of the total sample was 61 years; there were more female (*n* = 22/30, 73%) than male participants (*n* = 8/30, 27%). The mean hand pain score over the past 7 days on a 0–10 numerical rating scale at screening was 5.9 (range 4–9). There was representation across education levels and working status. The characteristics of the erosive and non-erosive samples were similar.

**Table 1:** Patient demographics and characteristics

Description	Erosive HOA (*N* = 15)	Non-erosive HOA (*N* = 15)	Total sample (*N* = 30)
Age, years, mean (min, max)	61 (43, 79)	56 (40, 75)	61 (40, 79)
Sex, n (%)			
Female	11 (73)	11 (73)	22 (73)
Male	4 (27)	4 (27)	8 (27)
Ethnicity, n (%)			
Non-Hispanic or Latino	11 (73)	13 (87)	24 (80)
Hispanic or Latino	2 (13)	2 (13)	4 (13)
Missing data	2 (13)	0 (0)	2 (7)
Race, n (%)			
Black/African American	7 (47)	6 (40)	13 (43)
White	6 (40)	7 (47)	13 (43)
Hispanic	1 (7)	2 (13)	3 (10)
Did not specify	1 (7)	0 (0)	1 (3)
Work status, n (%)			
Working full or part time	5 (33)	8 (53)	13 (43)
Full-time homemaker	5 (33)	1 (7)	6 (20)
Retired	3 (20)	3 (20)	6 (20)
Disability	2 (13)	3 (20)	5 (17)
Highest level of education, n (%)			
College or University	5 (33)	5 (33)	10 (33)
Some years at college	3 (20)	1 (7)	4 (13)
High school diploma or GED	5 (33)	5 (33)	10 (33)
Some high school	2 (13)	2 (13)	4 (13)
Certificate program	0 (0)	1 (7)	1 (3)
Grade school	0 (0)	1 (7)	1 (3)
Average hand pain severity over past 7 days, 0–10 NRS^a^, mean (min, max)	6.0 (5, 8)	5.9 (4, 9)	5.9 (4, 9)
Total MHQ score, mean (SD)^b^	70.0 (18.8)	69.6 (18.4)	69.8 (18.6)
Location of soft tissue swollen and tender PIP, n (%)^c^			
Thumb	13 (87)	11 (73)	24 (80)
Middle finger	10 (67)	14 (93)	24 (80)
Ring finger	7 (47)	10 (67)	17 (57)
Index finger	6 (40)	6 (40)	12 (40)
Little finger	6 (40)	2 (13)	8 (27)
Location of soft tissue swollen and tender DIP, n (%)^c^			
Middle finger	10 (67)	14 (93)	24 (80)
Index finger	8 (53)	10 (67)	18 (60)
Ring finger	4 (27)	6 (40)	10 (33)
Little finger	3 (20)	2 (13)	5 (17)
Location of soft tissue swollen and tender MCP, n (%)^c^			
Middle finger	12 (80)	14 (93)	26 (87)
Index finger	8 (53)	10 (67)	18 (60)
Ring finger	6 (40)	7 (47)	13 (43)
Little finger	4 (27)	4 (27)	8 (27)
None	4 (27)	0 (0)	4 (13)
Swollen and tender CMC, n (%)^d^			
Yes	11 (73)	12 (80)	23 (77)
No	2 (27)	2 (13)	6 (20)
Inflammation in the affected hand, n (%)			
Yes	15 (100)	7 (47)	22 (73)
No	0 (0)	8 (53)	8 (27)
Location of OA elsewhere, n (%)^c^			
Knee	10 (66)	11 (73)	21 (70)
Back	1 (6)	1 (6)	2 (7)
Ankles	2 (13)	0 (0)	2 (7)
Hip	1 (6)	0 (0)	1 (3)
Current treatments, n (%)			
Naproxen	7 (47)	9 (60)	16 (53)
Ibuprofen	8 (53)	2 (13)	10 (33)
Acetaminophen	3 (20)	0 (0)	4 (13)
Tramadol	0 (0)	3 (20)	2 (7)
Oxycodone	0 (0)	2 (13)	2 (7)
Hydrocodone	0 (0)	2 (13)	2 (7)
Voltaren gel	0 (0)	1 (6)	1 (3)
Meloxicam	0 (0)	1 (6)	1 (3)
Aspercream	0 (0)	1 (6)	1 (3)
Celecoxib	1 (6)	0 (0)	1 (3)
Duloxetine	1 (6)	0 (0)	1 (3)
Fish oil	1 (6)	0 (0)	1 (3)
Flexeril	0 (0)	1 (6)	1 (3)

*CMC* Carpometacarpal, *DIP* Distal interphalangeal, *GED* General Educational Development, *HOA* Hand osteoarthritis, *MCP* Metacarpophalangeal, *MHQ* Michigan Hand Outcomes Questionnaire, *NRS* Numerical rating scale, *OA* Osteoarthritis, *PIP* Proximal interphalangeal, *SD* Standard deviation

^a^0 = no pain, 10 = worst pain imaginable

^b^Data included for 27 out of the 30 participants, due to missing or incomplete data for 3 participants (*n* = 2 erosive, *n* = 1 non-erosive). The total MHQ score ranges from 0 to 100, with higher scores indicating better hand performance

^c^More than one option could be selected for each participant

^d^Missing data for one participant

Concept saturation was achieved in the combined erosive and non-erosive sample with no new relevant concepts emerging in the final set of concept elicitation interviews (Supplementary Figure S1). Concept saturation was also achieved for symptom concepts in the erosive and non-erosive sub-samples. Each of the sub-samples approached saturation for impact concepts with a small proportion of new sub-concepts emerging in the final sets of transcripts: hobbies (*n* = 1), pushing (*n* = 1) and bending (*n* = 2) in the erosive sample, and use of hand tools (*n* = 1) in the non-erosive sample.

### HOA symptoms

**Fig. 2:**
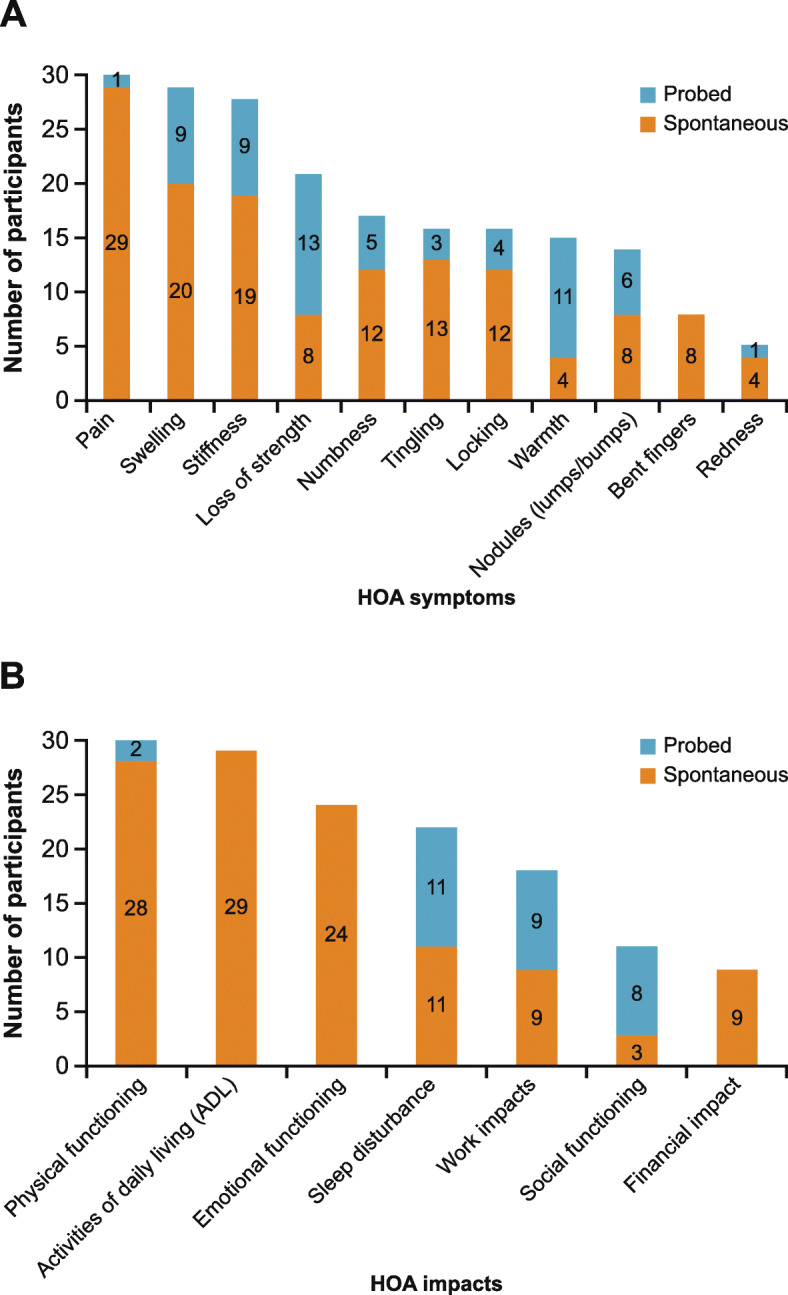
Overview of **a** HOA symptoms and **b** HOA impacts reported in concept elicitation interviews. HOA: Hand osteoarthritis

Eleven HOA symptoms were reported during the concept elicitation interviews (Fig. 2a**,** Table 2); the most frequently reported symptoms were pain (*n* = 30, 100%), swelling (*n* = 29, 97%) and stiffness (*n* = 28, 93%). With the exception of nodules (often described as ‘lumps’ or ‘bumps’), which were more frequently reported by those with erosive HOA (erosive HOA, *n* = 11; non-erosive HOA, *n* = 3), a similar number of erosive and non-erosive HOA participants reported each of the symptoms and impacts.

**Table 2:** Summary of symptoms reported in the concept elicitation interviews

Concept	Participants, n (%)		Example quote
	Erosive HOA sample (***N*** = 15)	Non-erosive HOA sample (***N*** = 15)	
Pain	15 (100)	15 (100)	*“It’s kind of like when any part of your body is sore, you know, you—or have a soreness kind of a feeling, it’s kind of that type of feeling but in your, in your hand. And that becomes noticeable, more noticeable when you move—try to make movement.”* (42-year-old female)
Swelling	14 (93)	15 (100)	*“Well it’s just swollen. It swells up around the joint and you can touch it and you can feel the swelling that’s there.”* (70-year-old female)
Stiffness	13 (87)	14 (93)	*“Like if sometimes when, um, you know like you try to close your hand... It’s stiff and sometimes it’s hard to close your hand all the way.”* (73-year-old female)
Loss of strength	10 (67)	11 (73)	*“I have to more and more use both hands because I don’t have the, um, same strength. And the pain causes like a lack of strength I guess you could say.”* (40-year-old female)
Numbness	8 (53)	9 (60)	*“Well I don’t know if you’ve ever experienced numbness, but it’s just like where I can’t feel how tight I’m gripping something or if I’m—really I can’t feel that I’m even holding it.”* (64-year-old male)
Tingling	7 (47)	9 (60)	*“Well I wake up, my hand is already tingling”* (44-year-old male)
Locking	7 (47)	9 (60)	*“I can’t pry them and sometimes I have to pry them loose and really, really force them loose like, you know, because it’s so locked in.”* (53-year-old female)
Feeling of warmth in hand(s) or finger(s)	6 (40)	9 (60)	*“where it’s painful and it’s, um, um, stiff and it’s throbbing and, you know, it’s painful to the touch also it feels to me anyway it feels more warm like, like, like as if it’s got a fever almost”*(40-year-old female)
Nodules (lumps/bumps)	11 (73)	3 (20)	*“Well it’s like on the tip of every finger it’s lumpy. It’s got lumps on it. You know, like lumps. And that is very painful.”* (64-year-old female)
Bent fingers	5 (33)	3 (20)	*“And the pointer finger will be that way also, but it is permanently or almost—not, not all the way bent, but it is starting to bend at the first knuckle.”* (53-year-old female)
Redness	2 (13)	3 (20)	*“It’s red on top, on the top of the knuckle. It’s like, like you hit it with a hammer or something.”* (66-year-old male)

*HOA* Hand osteoarthritis

In the symptom selection exercise, 22 participants reported their most bothersome symptoms, with pain (*n* = 18, 82%), stiffness (*n* = 17, 77%) and swelling (*n* = 11, 50%) most frequently selected. Fifteen participants reported the most important symptoms to treat, with stiffness (*n* = 14, 93%) and pain (*n* = 13, 87%), followed by swelling (*n* = 9, 60%) and limited ability to move (*n* = 9, 60%), most frequently selected.

Most participants suggested that many symptoms tended to occur together. Pain, swelling and stiffness were reported to be closely related: “*I would think that there is a relationship between swelling, stiffness, stiffness, and pain. Um, because when I, when I’m swollen and, and a little stiff, my discomfort is—I will feel more achy*” (42-year-old female).

#### Pain

Most participants indicated that they experienced pain on a continuous or daily basis (*n* = 16/28, 57%) or every other day (*n* = 9/28, 32%). In addition to the term ‘pain’, participants most commonly described their hands as feeling ‘tender’ (*n* = 17/30, 57%) or ‘aching’ (*n* = 13/30, 43%): *“uh, the pain is just an aching, dull pain that’s always there”* (59-year-old male). Approximately a quarter of the sample also referred to the pain as a ‘sharp’ or ‘stabbing’ pain, a ‘throbbing’ pain or ‘soreness’ (each *n* = 8/30, 27%). When describing their pain on a 0–10 scale (0 being ‘no pain’ and 10 being ‘pain as bad as you can imagine’), participants reported a mean pain severity of 5.4 at resting/normal and 8.2 at its worst.

#### Swelling

Participants indicated that they experienced swelling constantly (*n* = 10/23, 43%) or 1–3 times a week (*n* = 6/23, 26%). Almost all participants used the terms ‘swelling’ and ‘swollen’ (*n* = 28/29; 97%): *“oh, they just – just look very fat, swollen”* (55-year-old female). Most described the severity of their swelling as mild or moderate (*n* = 11/20, 55%). Several participants described their swelling to be between 5 and 8 on a 0–10 scale from ‘no swelling’ to ‘swelling as bad as you can imagine’ (*n* = 6/20, 30%), with one participant describing the swelling as ‘very severe’.

#### Stiffness

Several participants reported that they experienced stiffness constantly (*n* = 5/15, 33%) or every day (*n* = 3/15, 20%): “*no matter if it’s a severe day or a mild day, it’s stiff constantly—pretty much constantly unless I work my hand*” (44-year-old male). The most commonly used terms were ‘stiff’, ‘stiffness’ or ‘stiffening’ (*n* = 16/27, 59%). Most participants described the severity of stiffness to be between 7 and 10 on a 0–10 scale from ‘no stiffness’ to ‘stiffness as bad as you can imagine’ (*n* = 7/20, 35%). One participant described the stiffness as ‘quite severe’, suggesting it is among the most severe symptoms patients experience, as well as being among the most frequent.

### Impacts of HOA

During the concept elicitation interviews, participants discussed the impact of HOA on 7 high-level domains of functioning/HRQoL: physical functioning, emotional well-being, social functioning, ADL, sleep disturbance, work impacts and financial impacts (Fig. 2b, Table 3). A similar number of participants from the erosive and non-erosive samples discussed being impacted for each of the HRQoL domains. The way participants described their experience of impacts was also largely similar. Impacts on physical functioning and ADL were the most commonly reported (*n* = 30, 100%, and *n* = 29, 97%, respectively). In addition, impact on work is not commonly reported in the literature; thus, we focus on these three impacts below (data for all impact domains are presented in Table 3 and Supplementary Table S2); note, additional sub-concepts were discussed in the interviews.

**Table 3:** Summary of impacts reported in the concept elicitation interviews

Concept	Participants, n (%)		Example quote
	Erosive HOA sample (***N*** = 15)	Non-erosive HOA sample (***N*** = 15)	
Physical functioning	15 (100)	15 (100)	*“I can’t, like, open up a jar or pick up big objects with it. And, um, like I said, it’s - it’s just painful.”* (61-year-old female)
Activities of daily living	14 (93)	15 (100)	*”It’s just hard to button up things. You can’t hardly like zip up things. It’s just annoying.* “(62-year-old female)
Emotional functioning	13 (87)	11 (73)	*“annoying, because sometimes, you know, you can’t hardly grab things. You’re dropping things … it’s just aggravating when you can’t do just everything, things like you used to, it’s harder now.”* (62-year-old female)
Sleep disturbance	11 (73)	11 (73)	*“if I fall asleep on my hand or resting my head in my hand and I can feel it getting crampy and tight, it will wake me up and then I will kind of shake my hand out and move it to another position”* (57-year-old female)
Work impacts	8 (53)	10 (67)	*“I used to be a carpenter but, uh, I couldn’t hold a hammer. I couldn’t grip the, uh, the tools. And so, uh, uh, I had to, uh, stop, uh, doing the, the labor, labor part of the job”* (64-year-old male)
Social functioning	5 (33)	6 (40)	*“I haven’t went fishing with my friends in quite a while because, you know, trying to reel in the reel and everything … we don’t get to go out on the boat like we used to and have that one on one time or the three of us would go out, the three guys, and hang out.”* (44-year-old male)
Financial impact	5 (33)	4 (27)	*”Sometimes it do because of the money, you know, the extra medication, the arthritis—you know, Tylenol, arthritis medication, that’s more money. I have to buy more, you know, I have to buy more, more pills, more medication for that purpose”* (53-year-old female)

*HOA* Hand osteoarthritis

#### Physical functioning

Difficulties picking up/lifting objects (*n* = 26, 87%), gripping objects (*n* = 25, 83%) and carrying/holding objects (*n* = 21, 70%) were most frequently reported, while bending (*n* = 5, 17%), fine motor movements (*n* = 2, 7%), pushing (*n* = 1, 3%) and squeezing (*n* = 1, 3%) were mentioned to a lesser extent. The impacts were frequently attributed to pain (*n* = 16 times the impact was attributed to the symptom overall [this is not the total number of participants who attributed a symptom to impacts on physical functioning]), loss of strength (*n* = 9) and swelling (*n* = 8), and to a lesser extent numbness (*n* = 4) and stiffness (*n* = 4).

#### ADL

Difficulties with housework (*n* = 24, 83%) and getting dressed (*n* = 22, 76%) were the most frequently reported impacts on ADLs. Participants also reported impacts on electronic device use (*n* = 20, 69%), cooking and preparing food (*n* = 19, 66%), writing due to difficulties holding and/or gripping the pen/pencil (*n* = 17, 59%), washing/general self-care tasks (*n* = 14, 48%) and driving (*n* = 14, 48%). The impacts on ADLs were most frequently attributed to pain (*n* = 57 times the impact was attributed to pain symptoms overall), followed by loss of strength (*n* = 17), swelling (*n* = 11) and numbness (*n* = 11). Note, some patients attributed a symptom to impacting on more than one ADL.

#### Work impacts

Although 43% (*n* = 13) of participants were currently employed full or part time (Table 1), 60% (*n* = 18) were able to comment on impacts experienced during previous or current work as a result of their HOA. Participants working in both manual and office environments described having work limitations due to their HOA. Participants discussed difficulties typing (*n* = 5, 28%), writing (*n* = 5, 28%), picking and lifting objects (*n* = 5, 28%), carrying, holding or gripping objects (*n* = 4, 22%) and having the need to stretch or exercise the joints while at work (*n* = 3, 17%). Thirteen of the 18 (72%) participants that described work impacts stated that there had been no change to their work status; others reported that they were not working due to HOA (*n* = 2, 11%), working less/fewer hours due to their HOA (*n* = 1, 6%) and were both working less and stopped the physical labor aspect of their job because of HOA (*n* = 1, 6%). Of the 9 participants who reported financial impacts, 6 (67%) talked about their impaired ability to work due to HOA negatively affecting their financial situation.

### RTDC app findings

A total of 10 participants (erosive: *n* = 5; non-erosive: *n* = 5) took part in the RTDC app activity over a 7-day period. Most participants had moderate–high adherence to the daily symptom ratings and 14 qualitative tasks over the 7 days. Over half (*n* = 7/10, 70%) of participants completed at least 86% of the ratings and tasks. The remaining three participants had completion rates of 49%, 66% and 69%. RTDC identified the same core symptoms (pain, stiffness and swelling) and impact domains as reported in the concept elicitation interviews. However, 6 (60%) participants discussed experiencing symptoms and/or impacts of HOA they had not mentioned in their interview, but that had already been elicited via other participants’ interviews; of these, the impacts were reported in the ADL (*n* = 9), physical functioning (*n* = 2), emotional functioning (*n* = 1), sleep disturbance (*n* = 1) and work (*n* = 1) domains. Two new ADL impact concepts were identified through RTDC that had not been reported in any interviews: tying things (*n* = 5) and turning a key in a lock (*n* = 1).

### Treatment experience of HOA

As reported in Table 1, participants used a range of pain relief medications. Twenty participants rated their satisfaction with current treatment on a scale of 0 (very dissatisfied) to 5 (very satisfied); overall, participants had a moderate level of satisfaction with their treatment (mean rating 3.7). The mean satisfaction rating given for over-the-counter analgesics was 3.6 (*n* = 23), whilst for the 5 patients receiving prescription opioids, mean treatment satisfaction was 3.8 (*n* = 6). Of the 19 participants who described their overall level of satisfaction with their current treatment’s effects on symptoms, 12 (63%) discussed feeling satisfied with the relief experienced. Of the 8 participants who described their level of satisfaction with duration of relief, 5 (56%) were dissatisfied, explaining that it was temporary/short-lived and that they wished the relief would last longer; the remaining 3 participants were satisfied. Of the 11 participants who commented on their satisfaction with mode of administration, 9 (82%) were satisfied, all of whom received oral treatment (Supplementary Table S3). Of the two who were not satisfied, one participant said that they often forgot to take their oral treatment, and the other who was using Salonpas patches was satisfied with ease of administration but was dissatisfied with the smell of the treatment. Most participants (20/30, 67%) reported that their treatment goal was to improve symptoms, with a focus on reducing/relieving pain (Supplementary Figure S2). Several participants (13/30, 43%) also stated goals related to physical functioning. Other goals included a cure for HOA (*n* = 5), maintaining/preventing worsening symptoms (*n* = 2), greater duration of relief (*n* = 1), avoidance of stronger treatments (*n* = 1) and a treatment that is “good for their body” (*n* = 1). Of the 18 participants who discussed whether their current treatment allowed them to meet their goals, 11 (61%) stated that it did not (Supplementary Table S3).

### Conceptual model

**Fig. 3:**
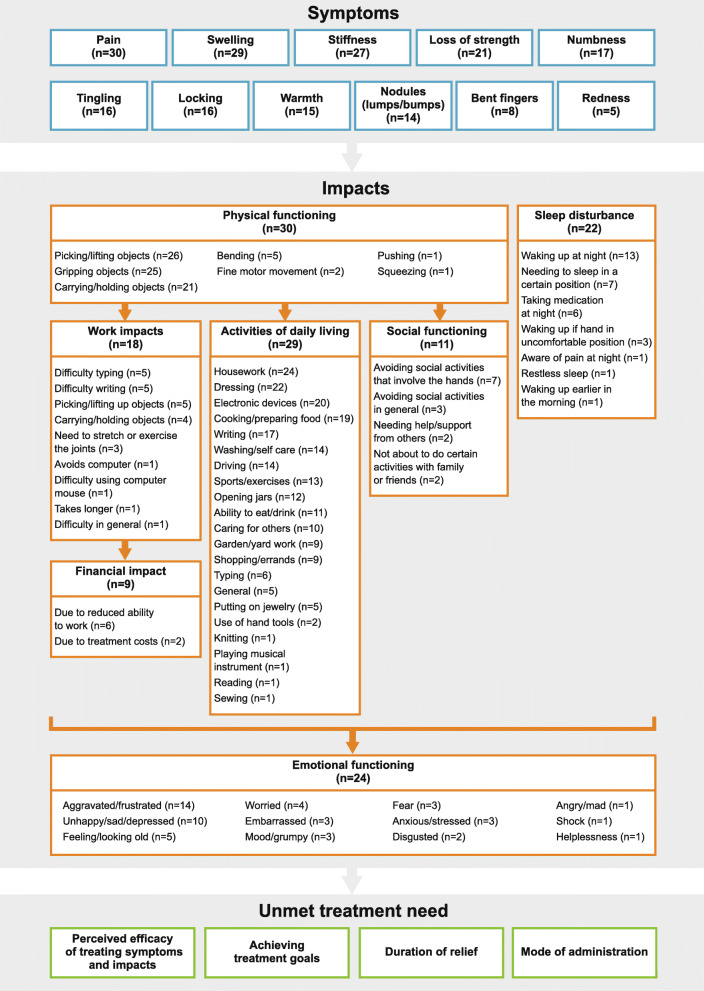
Conceptual model

The conceptual model, developed based on the concept elicitation interview results, illustrates the symptoms and impacts (concepts) relevant to patients with HOA (Fig. 3). Eleven key symptoms of HOA were identified, along with seven core categories of functional impacts. The conceptual model also illustrates the causal relationships between the concepts, as established through the qualitative interviews. Indeed, the disease symptoms experienced by patients can cause impacts to their quality of life; for example, swelling and stiffness may cause impacts to physical functioning, which in turn, have an impact on their ability to work. The seven functional impacts can also cause impacts to their emotional functioning. In representing the various concepts relevant in the disease, the conceptual model can also aid in highlighting targets for treatment. Four unmet treatment needs were identified: perceived efficacy of treating symptoms and impacts, achieving treatment goals, duration of relief, and mode of administration.

### Concept mapping and MHQ cognitive interview

While several prominent and important symptoms reported in the interviews are captured in the MHQ (i.e. joint pain, loss of strength and numbness), neither stiffness nor swelling are captured. The MHQ also captures additional HRQoL impacts to those reported by participants. MHQ domains include relevant aspects of physical functioning, limitations in ADL, emotional/psychological functioning, and the additional impacts limitations in role (work/school), and in social/leisure activities.

Participants were asked to provide feedback on each of the 37 MHQ questions in order to establish level of understanding of each item (eg, by explaining the question in their own words). In the cognitive interviews, most participants asked found the MHQ items easy to understand (*n* = 22/24, 92%) and the instructions easy to follow (*n* = 21/23, 91%) (Supplementary Figure S3A). A small number of participants did not demonstrate understanding of the item asking about ‘accomplishment at work’ (*n* = 3/30, 10%), and the item asking about ‘satisfaction with feeling (sensation)’ appeared ambiguous, with 2/30 (7%) participants reflecting on a range of sensations. The response options were reported as clear and easy to use by most participants asked (20/27 (74%). Participants understood and were generally satisfied with the recall periods of ‘during the past week’ and ‘during the past 4 weeks’, with 92% and 70% of participants reporting them as “reasonable”, respectively. When assessing the items of the MHQ, a small number of participants did not use the appropriate recall period. For example, in Part I (functioning of the hand(s)/wrist(s) during the past week), five participants thought back longer than a week, and in Part II (ability of the hand(s) to do certain tasks during the past week) seven participants used a different recall period than instructed. However, only one participant used the incorrect recall period throughout the whole interview.

In addition to assessing understanding of the MHQ items, participants were also asked to provide feedback on the relevance of the individual items to their experience of HOA. The majority of MHQ items were relevant to participants’ experience of HOA (Supplementary Figure S3B), with no distinguishable differences between erosive and non-erosive subgroups. Items asking about the impact of the appearance of the hands were the least relevant, with only 8/29 participants (27.6%) deeming “the appearance of my right/left hand interfered with my normal social activities” as relevant and 9/29 (31%) deeming “the appearance of my right/left hand made me depressed” as relevant. Most participants asked (*n* = 23/27, 85%) reported that there were no concepts missing, while missing concepts related to burning/tingling sensations, consideration of the weather and time of year, numbness and dropping objects, and the least/most painful time were reported by 1 (3%) participant each. Stiffness and swelling are also missing from the MHQ. Despite these symptoms being frequently reported by participants in the concept elicitation interviews, no participants reported these symptoms as missing.

## Discussion

This study aimed to add to the body of evidence surrounding the symptoms, HRQoL impacts and treatment experiences of patients with HOA, and to explore any differences between patients with erosive and non-erosive HOA. It was also designed to assess content validity of the MHQ in a HOA population.

The experience of patients with erosive and non-erosive HOA was found to be largely similar, with the same symptoms and impacts being reported by each group, and by similar numbers of patients in most cases. Pain, swelling and stiffness were the most frequently reported and most bothersome symptoms for both erosive and non-erosive HOA groups, highlighting them as important concepts to the HOA experience. Other symptoms included loss of strength, numbness, tingling, locking and a feeling of warmth in their hand(s) or finger(s). Generally, participants did not recognize their symptoms as occurring individually, but rather described them occurring collectively; in particular, pain, swelling and stiffness were suggested to be closely related.

Pain, swelling, stiffness and loss of strength, were the symptoms most commonly described as having a direct impact on physical functioning in both erosive and non-erosive groups; these in turn impacted ADLs, emotional well-being, sleep, social functioning and finances. For both groups, HOA was reported to have an impact on both work productivity and ability to work in paid employment, with 22% of those who described work impacts reporting that they were no longer working, were working fewer hours, or had dropped labor aspects of their job due to HOA. Given that the symptoms and impacts of HOA are closely interlinked, working to alleviate the symptom burden of HOA would likely improve physical functioning and reduce disability, which may in turn have positive effects on patients’ ability to work or work full time, and overall HRQoL.

These findings are consistent with previous studies. Thumboo et al., found pain, stiffness, swelling, and functional impacts such as problems carrying or gripping items, as well as emotional and social impacts were important to Asian patients with symptomatic HOA [36]. Studies in European patients found similar impacts of HOA on hand mobility and grip force, ADLs, work status, social life/participation and emotional state (e.g. feeling embarrassed or dependent on others) [37, 38].

There is a lack of literature that differentiates or compares the patient experience between erosive and non-erosive HOA. Our findings show the experience of erosive and non-erosive HOA is largely similar, but there may be differences (specifically in the frequency that nodules were reported). Importantly, each symptom and impact identified was reported by patients with erosive and non-erosive HOA, providing evidence that patients experience these different disease sub-types in a similar way. Further supporting this is that the number of patients in each sub-group reporting each symptom and impact was similar. The exception to this was nodules, which were reported more frequently by those with erosive HOA than non-erosive HOA. Consistently, one study found that patients with erosive HOA had more nodes and report more esthetic damage than patients with non-erosive HOA [10, 39]. Previous studies have also shown that pain is more severe and there is more functional impairment with erosive HOA [8, 9, 40], whereas our qualitative study demonstrated no obvious differences between the two sub-samples. Conversely, a prospective cross-sectional study found no significant difference in pain at rest between patients with erosive and non-erosive HOA [10]. These findings highlight the need for further research to understand if there are differences in the severity of symptoms and impact on HRQoL experienced by patients with erosive versus non-erosive HOA; this could be explored using valid PRO instruments in a larger sample.

Treatment options for HOA are limited, with most targeting symptomatic and functional improvement, rather than treating the underlying disease process that causes structural damage of the joint [41, 42]. In this study, participants reported that their current treatments (mainly analgesics, including anti-inflammatory medications) led to a reduction in pain, swelling and stiffness that was often short-lived. Together, these findings highlight an unmet need for disease-modifying HOA treatments with improved efficacy and durability of symptom relief.

Concept elicitation interviews remain the gold standard for providing in-depth understanding of patient experience. However, they are typically conducted in a formal or non-familiar environment, which can make participation difficult for some individuals, and rely on participants’ memory, possibly introducing recall bias. In the present study the interviews were conducted by telephone, which has the advantage of being more convenient for participants and facilitates the inclusion of geographically dispersed participants and those who might have difficulty (for health or practical reasons) traveling to an interview facility. Telephone interviews can also help participants to feel more confident and at ease than they might feel in more formal and less familiar settings. However, limitations of telephone interviewing in comparison with face-to-face interviews include not being able to observe facial expressions and respond to non-verbal cues, and there are fewer opportunities for building rapport between the participant and interviewer. A literature review assessing telephone versus face-to-face interviews in 15 studies from the peer-reviewed literature suggested that while the lack of non-verbal communication associated with telephone interviews may be a slight disadvantage for cognitive debriefing, telephone interviews are well suited to concept elicitation, and overall, little to no data quality is lost [43].

Advances in technology may provide a way to overcome the limitations of formal concept elicitation interviews in general (both telephone and face-to-face), such as through using RTDC methods employed here, which may strengthen ecological validity by capturing data ‘in the moment’ as participants go about their daily lives. This novel approach is less reliant on accurate recall by patients, and it is possible that some patients may find the process less stressful because they are in a more comfortable environment, can respond at a time of their own choosing, can take as long as they need and are not interacting directly with a person, perhaps resulting in more candid answers. Furthermore, everyday experiences may remind patients of important impacts that they may not recall during a formal interview.

The RTDC results in this study supported the overall findings of the concept elicitation interviews and revealed two novel impact concepts not reported in any interviews. In addition, a number of participants described symptoms and impacts that they had not shared during their interviews but that had been reported in other interviews. Similarly, a study that evaluated concept elicitation interviews, social media review and online group concept mapping found these concept elicitation interviews achieved an in-depth understanding of patient experience, but the novel methods identified different concepts and provided complementary concept elicitation approaches [44]. Therefore, using a range of complementary methods may help elucidate a full picture of the patient experience. Further research into using apps for collecting patient experience data would help to better understand these potential benefits.

Patient-centric research with qualitative interviews is important to inform trial design and drug development strategies, and for selecting outcome instruments that assess concepts that are important to patients. Here, we assessed the suitability of the MHQ as an endpoint in HOA clinical trials to assess the symptoms and impacts of HOA. Although the AUSCAN also measures these key symptoms [17], this study focussed on the MHQ because of its greater number of concepts assessed and potentially higher sensitivity. Our findings provide evidence that the MHQ assesses many relevant concepts and is clear and easy for patients of varying education levels to understand. However, we did identify a small number of MHQ items which some participants did not understand or viewed as ambiguous; minor edits to the wording of these items may help to improve the MHQ. Any suggested revisions are deemed non-essential, as the instrument would likely be acceptable in its current form, and revising the MHQ was outside of the scope of the current study. With the addition of items assessing stiffness and swelling, the findings support use of the MHQ in an HOA population to evaluate the most relevant domains of HRQoL in clinical trials and other research studies. The addition of stiffness and swelling items to the MHQ would require cognitive and psychometric evaluation prior to use as an endpoint in clinical trials; alternatively, a separate instrument for stiffness and swelling could be used alongside the MHQ to capture these concepts.

A limitation of this study was that the research was performed only in the United States; therefore, further study in other countries would be valuable to confirm generalizability of the findings, particularly in relation to relevant HRQoL domains, which are more likely to vary by culture than symptoms [45]. Although quotas were used to ensure that a diverse sample of patients was recruited in terms of age, highest education level, ethnicity and race, the sample included no representation of patients with milder pain (pain rating < 4), and, the non-erosive group only included patients who were not taking or had disease inadequately controlled by non-opiate analgesics. This lack of diversity was based on the necessity to recruit patients experiencing symptoms in order to characterize the disease experience and the impact of symptoms in HOA, which was the primary aim of this study. It did, however, introduce a selection bias, and thus, the results are representative of a specific population. The generalizability of these results is further limited by the finding that 77% of all participants experienced swelling and tenderness of the carpometacarpal joint; a location at which osteoarthritis is associated more with pain and disability than the interphalangeal joints [46]. A further limitation of this study is the difficulty in confirming that all symptoms are caused by the patient’s HOA. Whilst none of the sample had diagnoses of comorbidities such as carpal tunnel or fibromyalgia that may have symptoms of tingling and numbness, it is possible that they were present but undiagnosed. However, as there is evidence for a possible neuropathic component to HOA [47], patients with these symptoms were still included in the sample.

In qualitative research aimed to explore phenomena, sample sizes are determined based on concept saturation. Saturation can be achieved in as few as 12 individual interviews in a relatively homogenous population [35, 48]. Concept saturation is a widely accepted method for guiding sample size in qualitative research [30, 49]. In this study, although the sample size was small, it was deemed sufficient to achieve the study aims. That is, the combined erosive and non-erosive HOA sample reached concept saturation, with the sub-groups achieving saturation for symptoms, and nearly achieving saturation for impacts. Based on the finding that the disease experience of the erosive and non-erosive HOA sub-groups appears to be very similar (with the exception of the reported frequency of nodules), the concept saturation results confirm the adequacy of the sample size of *n* = 15 in each sub-group and *n* = 30 overall to achieve the aims of this qualitative study.

## Conclusions

This study provides evidence that the key symptoms of HOA (pain, swelling and stiffness) have an impact on several domains of HRQoL, and are not well-controlled by currently available therapies in this targeted population of individuals erosive and non-erosive HOA, thus confirming an unmet treatment need for more effective, durable, and disease-modifying therapies. Importantly, the disease experience was found to be similar in the HOA sub-types, with a similar number of patients in the erosive and non-erosive HOA groups reporting each symptom and impact, except for nodules, which were reported more commonly by those with erosive HOA. Furthermore, all of the symptoms and impacts were also described in a similar way by the erosive and non-erosive groups, suggesting that patients’ experiences are similar.

Although the sample was targeted, and thus not necessarily representative of the broader population of erosive and non-erosive HOA, both sub-types approached concept saturation. These exploratory findings that patients with erosive and non-erosive HOA experience the disease similarly can help to inform the PRO measurement and endpoint strategy for future HOA studies. RTDC findings support its potential as a valuable and feasible approach for generating patient insights to supplement traditional concept elicitation interview methodology, potentially providing a more complete picture of an individual’s disease experience. The cognitive interview findings provide partial support for the suitability of the MHQ as an assessment of HRQoL in a HOA population. The MHQ could be used as a comprehensive measure of HRQoL in patients with HOA with the addition of items assessing stiffness and swelling. Alternatively, separate patient-reported items to assess stiffness and swelling in each hand could be used in conjunction with the MHQ, to ensure all symptoms important to patients with HOA are assessed.

## Supplementary Information

**Additional file 1 **: **Supplementary Materials**. **Supplementary Table S1**. Description of RTDC qualitative tasks. **Supplementary Table S2**. Summary of impact concepts and sub-concepts reported in the CE interviews. **Supplementary Table S3**. Current treatment satisfaction and treatment goals, with example participant quotes. **Supplementary Fig. S1**. Symptom and impact level saturation analysis (total sample). **Supplementary Fig. S2**. An overview of the reported goals for treatment. **Supplementary Fig. S3**. Cognitive interview results showing (A) understanding and (B) relevance for MHQ items.

## Data Availability

GlaxoSmithKline (GSK) makes available anonymized individual participant data and associated documents from interventional clinical studies which evaluate medicines, upon approval of proposals submitted to www.clinicalstudydatarequest.com. To access data for other types of GSK sponsored research, for study documents without patient-level data and for clinical studies not listed, please submit an enquiry via the website.

## Abbreviations

ADL: Activities of daily living
AUSCAN: Australian Canadian Osteoarthritis Hand Index
CMC: Carpometacarpal
DIP: Distal interphalangeal
GED: General Educational Development
HOA: Hand osteoarthritis
HRQoL: Health-related quality of life
MCP: Metacarpophalangeal
MHQ: Michigan Hand Outcomes Questionnaire
OMERACT: Outcome Measures in Rheumatology
PIP: Proximal interphalangeal
PRO: Patient-reported outcome
RTDC: Real-time data capture
SD: Standard Deviation
