# Supplementary material for: A qualitative exploration of the patient experience of erosive and non-erosive hand osteoarthritis
Source: J Patient Rep Outcomes. 2021 Feb 3;5:18. doi: 10.1186/s41687-021-00286-1 (PMC7859145; doi:10.1186/s41687-021-00286-1)
Supplement: Supplementary file 1 — Additional file 1 : Supplementary Materials. Supplementary Table S1. Description of RTDC qualitative tasks. Supplementary Table S2. Summary of impact concepts and sub-concepts reported in the CE interviews. Supplementary Table S3. Current treatment satisfaction and treatment goals, with example participant quotes. Supplementary Fig. S1. Symptom and impact level saturation analysis (total sample). Supplementary Fig. S2. An overview of the reported goals for treatment. Supplementary Fig. S3. Cognitive interview results showing (A) understanding and (B) relevance for MHQ items. [file 41687_2021_286_MOESM1_ESM.zip › HOA 206599_JPRO_Suppl_Revised 2_D1-clean.docx]

# Supplementary Materials

## Study population

Eligible participants (male or female) were 40–80 years of age; met American College of Rheumatology classification of hand osteoarthritis (HOA) [1]; a self-reported average hand-pain intensity over the past 7 days of ≥4 on a 0–10 numerical rating scale (NRS); negative titre rheumatoid factor and anti-**cyclic citrullinated peptide** antibody; had a personally signed written consent form; was verbally fluent and literate in English; and was able to attend two 45-minute telephone interviews (either the same day or on separate days). To be eligible for the erosive sample, participants were required to have imaging evidence of erosive disease on at least one proximal interphalangeal (PIP) or one distal interphalangeal (DIP) joint and to have active disease in at least one hand, with at least two soft tissue swollen and tender PIP and/or DIP joints in the affected hand, by clinical examination within the past 3 months. To be eligible for the non-erosive sample, participants were required to have at least one finger joint (DIP or PIP) with Kellgren–Lawrence (K-L) ≥2 [2] by X-ray in the last 12 months and be unwilling or unable to take non-opiate analgesics or was inadequately controlled by non-opiate analgesic.

Exclusion criteria included: history of any clinically significant inflammatory disease other than inflammatory osteoarthritis, especially, but not limited to, rheumatoid arthritis or spondyloarthropathies; diagnosis of rheumatoid arthritis, fibromyalgia, gout, calcium pyrophosphate deposition disease (CPPD), pseudogout, hemochromatosis or other inflammatory rheumatological or autoimmune disorders; clinical suspicion of, or previous investigation for CPPD or pseudogout, or history of chondrocalcinosis; any injury, medical or surgical procedure to the affected joint(s) that may interfere with evaluation of the target HOA joint(s); great difficulty hearing, reading or speaking; an uncontrolled psychiatric condition (e.g., schizophrenia, bipolar disorder) or mental condition rendering the patient unable to understand the nature, scope, and possible consequences of the study or likely to have difficulty participating in two 45-minute interviews; severe physical, neurological or cognitive deficits that might prohibit the ability to participate in an interview about the experience of their HOA and completion of a questionnaire; currently or has previously been enrolled in a clinical trial for OA in the past 6 months.

Participants who met inclusion/exclusion criteria were asked to complete a demographics form and an interview was scheduled for a mutually convenient time. Data was not collected from patients who declined to participate.

To take part in the real-time data capture, participants were also required to have access to a smartphone or tablet, be willing to take part in the app activities over the course of 7 days, and feel comfortable providing short videos or audio commentary and photos.

## References

1. Altman R et al. (1990) The American College of Rheumatology criteria for the classification and reporting of osteoarthritis of the hand. Arthritis Rheum 33:1601-1610.

2. Kellgren JH, Lawrence JS (1957) Radiological assessment of osteo-arthrosis. Ann Rheum Dis 16:494-502.

**Supplementary Table S1** Description of RTDC qualitative tasks

| Topic | Question |
| --- | --- |
| General experience | Describe in a video/audio recording how having arthritis in your hand(s) affects you day to day when you are in your home. |
| How stiffness compares from morning to evening | [MORNING] Describe in a video/audio recording any stiffness you have in your hand(s) this morning.  [EVENING] Describe in a video/audio recording any stiffness you have in your hand(s) this evening and how it compares to this morning. |
| How stiffness changes throughout the day | [EVENING] Please tell us in a short audio recording about how your stiffness changed throughout today. Did it feel the same all day, or did it get better or worse at different times of day? |
| Devices or aids | Take photos/videos of any aids, gadgets or devices that you use to help you to do certain activities or tasks. Please tell us in a recording about how they help you and the things you have difficulty doing without them. |
| Help from others | Tell us in a video/audio recording about any activities or tasks that you need help from someone else with because of the arthritis in your hand(s). |
| Impact on sleep | [MORNING] Tell us in a short video/audio recording about your sleep last night, and if it was affected by the arthritis in  your hand(s). |
| Impact on dressing and self-care | [MORNING] Describe in a video/audio recording any ways the arthritis in your hand(s) affects you when getting ready in  the morning. |
| Impact on preparing food/eating | Describe or show us in a video/audio recording any ways the arthritis in your hand(s) affects you when preparing food  or eating. |
| Work | Record any ways your work is affected by the arthritis in your hand(s). If you are not currently working in paid employment, tell us about how you are impacted at a regular activity you do inside or outside the home. |
| Impact of osteoarthritis in other bodily locations | Do you have arthritis anywhere else in your body, other than your hand(s)? Yes/No [PARTICIPANTS WHO RESPOND YES ONLY] Describe in a video/audio recording any ways the arthritis in the rest of your body compares to the arthritis in your hands. |
| Impacts in general | Describe in a video/audio recording the biggest challenge that you faced this week due to the arthritis in your hand(s) and how it made you feel. |
| Additional information | Thank you for taking part in these app tasks, if there is anything further you would like to tell us about the arthritis in your hand(s), please do so by text or audio recording. |

RTDC, real-time data capture.

**Supplementary Table S2** Summary of impact concepts and sub-concepts reported in the CE interviews

| Impact | Participants, n (%) |
| --- | --- |
| Physical functioning | 30 (100) |
| Picking up/lifting objects  Gripping objects  Carrying/holding objects  Bending  Fine motor movements  Pushing  Squeezing | 26 (87)  25 (83)  21 (70)  5 (17)  2 (7)  1 (3)  1 (3) |
| Activities of daily living | 29 (97) |
| Household maintenance  Housework  Cooking/preparing food  Opening jars/bottles  Gardening  Use of hand tools  Changing car tire  Self-care  Getting dressed  Washing/general self-care  Ability to eat/drink  Putting on jewelry  Use of electronics/communication  Use of electronic devices  Writing  Typing  Sports/exercise  Driving  Caring for others  Shopping/errands  General activities of daily living  Hobbies  Knitting  Playing musical instruments  Reading  Sewing | 24 (83)  19 (66)  12 (41)  9 (31)  2 (7)  1 (3)  22 (76)  14 (48)  11 (38)  5 (17)  20 (69)  17 (59)  6 (21)  13 (45)  14 (48)  10 (34)  9 (31)  5 (17)  1 (3)  1 (3)  1 (3)  1 (3) |
| Emotional functioning | 24 (80) |
| Aggravated/frustrated  Unhappy/sad/depressed  Feeling/looking old  Worried  Embarrassed  Mood/grumpy  Fear  Anxious/stressed  Disgusted  Angry/mad  Shock  Helplessness | 14 (58)  10 (42)  5 (21)  4 (17)  3 (13)  3 (13)  3 (13)  3 (13)  2 (8)  1 (4)  1 (4)  1 (4) |
| Sleep disturbance | 22 (73) |
| Work impacts | 22 (73) |
| Social functioning | 11 (37) |
| Financial impact | 9 (30) |

**Supplementary Table S3** Current treatment satisfaction and treatment goals, with example participant quotes

| **Category** | **Participants, n (N=30)** | **Response, n (%)** | **Example quote** |
| --- | --- | --- | --- |
| Effect on symptoms | 19 | Satisfied: 12 (63) | *“It kind of reduces the swelling, and, uh, you know, and of course, it reduces the pain, too.”* (53-year-old female) |
| Enablement of daily activities | 12 | Helpful: 8 (67) | *“Um, I’m satisfied so far, um, with it, um, because it’s helping me be able to work.”* (55-year-old female) |
| Mode of administration | 11 | Satisfied: 9 (82) | *“I’m okay with swallowing pills so that doesn't bother me at all.”* (59-year-old female) |
| Duration of relief | 8 | Satisfied: 3 (33) | *“That Aleve, you know, it'll go away for a few hours or so and uh, and it come back, you know?”* (73-year-old male) |
| Treatment goals | 18 | Not being met: 11 (61) | *“It doesn’t take away anything. It doesn’t take away all the pain. It doesn’t get rid of the stiffness. It doesn’t get rid of the numbness or the tingling. It is a thing that makes things a little more comfortable.”* (79-year-old female) |
|  |  |  |  |

Note: participants engaged in open-ended discussion about their treatment experience and did not have set options

**Supplementary Fig. S1** Symptom and impact level saturation analysis (total sample)


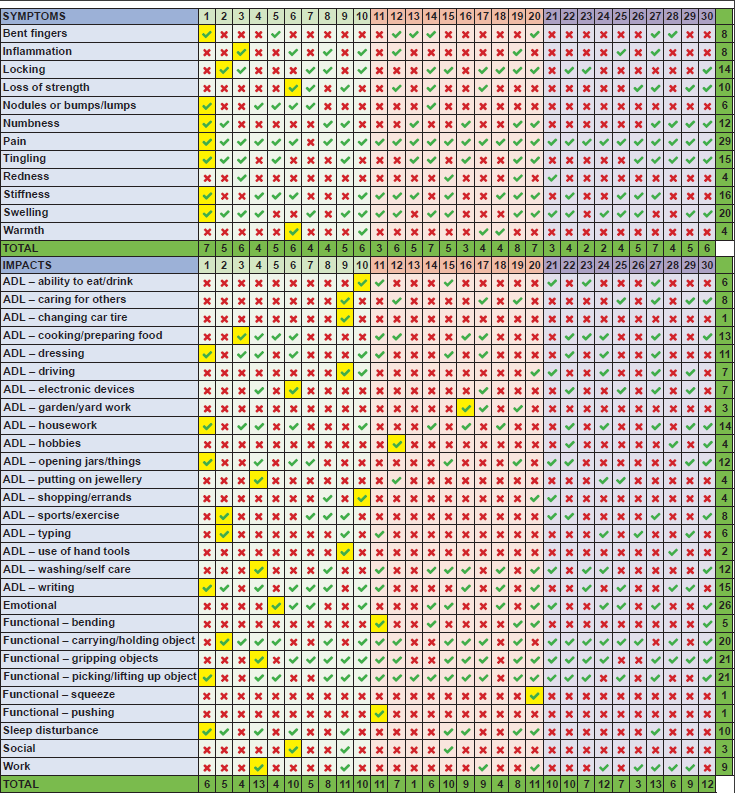
ADL, activities of daily living.

The cells highlighted in yellow indicate the first reporting of each concept. The total column indicates the number of participants that report each concept and the total row indicates the number of concepts reported for each participant.

**Supplementary Fig. S2** An overview of the reported goals for treatment


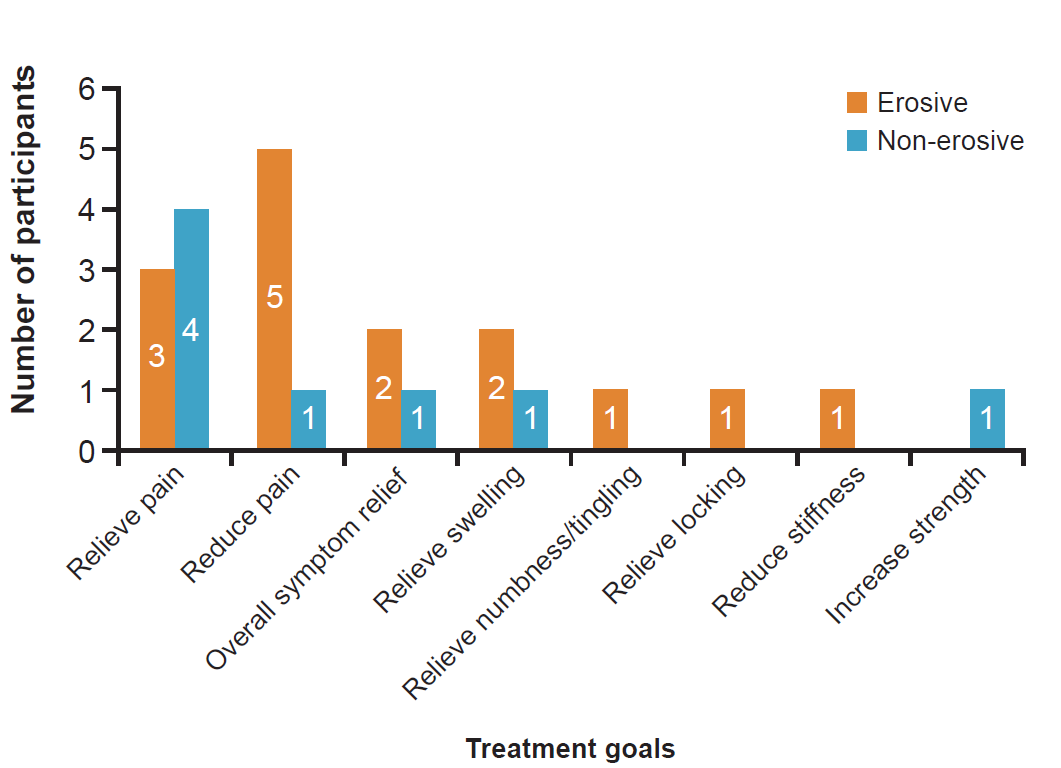


**Supplementary Fig. S3** Cognitive interview results showing (A) understanding and (B) relevance for MHQ items


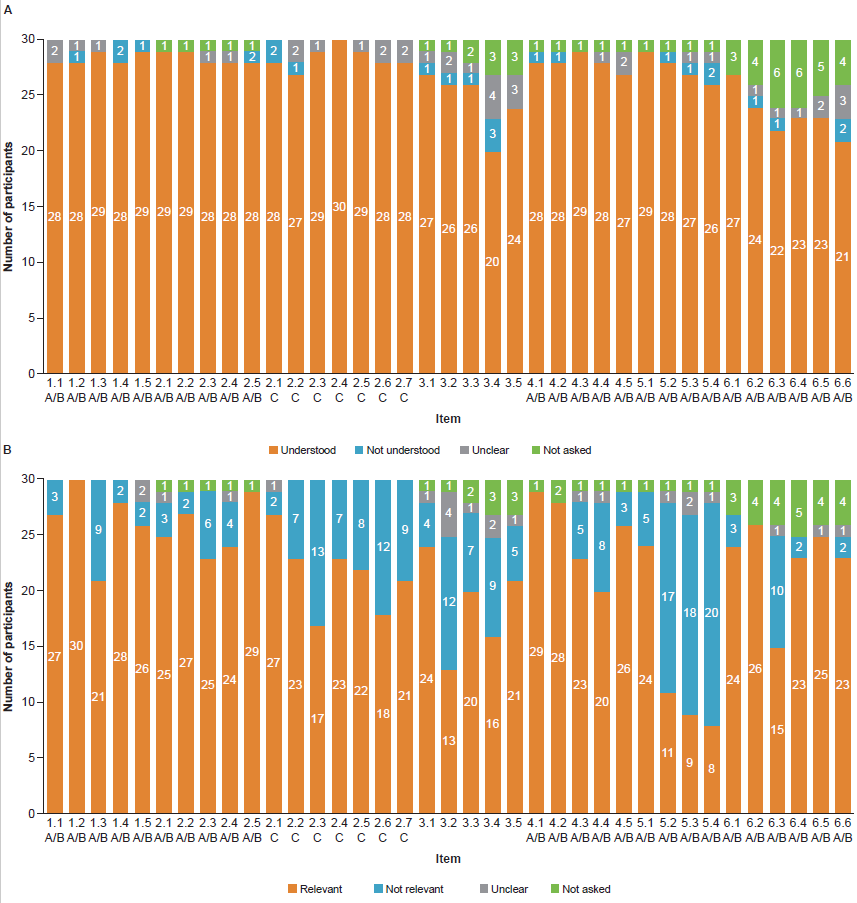


Note: Each column represents an individual item from the MHQ, with the (A) level of understanding, and (B) level of relevance associated with each item presented (based on feedback from participants).

In the MHQ, Part 1 assesses the functioning of the hand(s)/wrist(s) during the past week; Part 2 assesses the ability of the hand(s) to do certain tasks during the past week; Part 3 assesses the degree of impact on normal work (including both housework and school work) during the past 4 weeks; Part 4 assesses how much pain is experienced in the hand(s)/wrist(s) during the past week; Part 5 assesses the degree of impact as a result of the appearance of the hand(s) during the past week; and Part 6 assesses the level of satisfaction with the hand(s)/wrist(s) during the past week.

In Parts 1, 4, 5 and 6, Section A referred to the **right** hand/wrist and Section B referred to the **left** hand/wrist. As the items are otherwise the same, the level of understanding/relevance for the items across Section A and B have been assessed and presented together. In Part 2, Section C referred to **both hands**.
