# Supplementary figures and images for: A qualitative exploration of the patient experience of erosive and non-erosive hand osteoarthritis
Source: J Patient Rep Outcomes. 2021 Feb 3;5:18. doi: 10.1186/s41687-021-00286-1 (PMC7859145; doi:10.1186/s41687-021-00286-1)

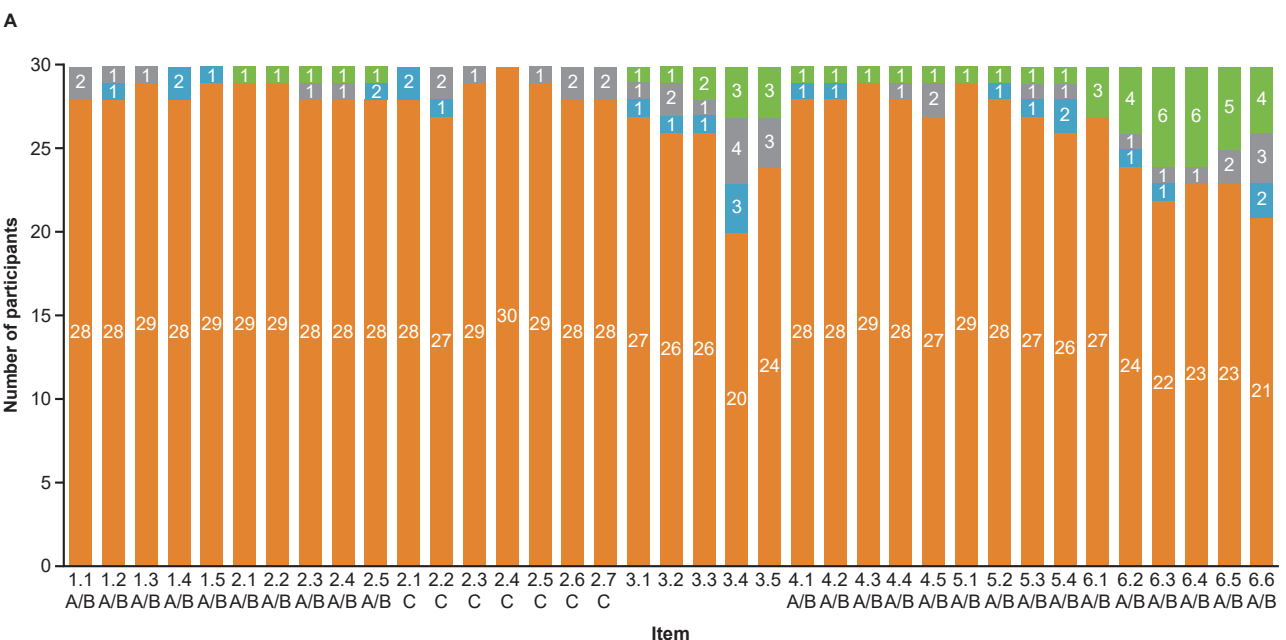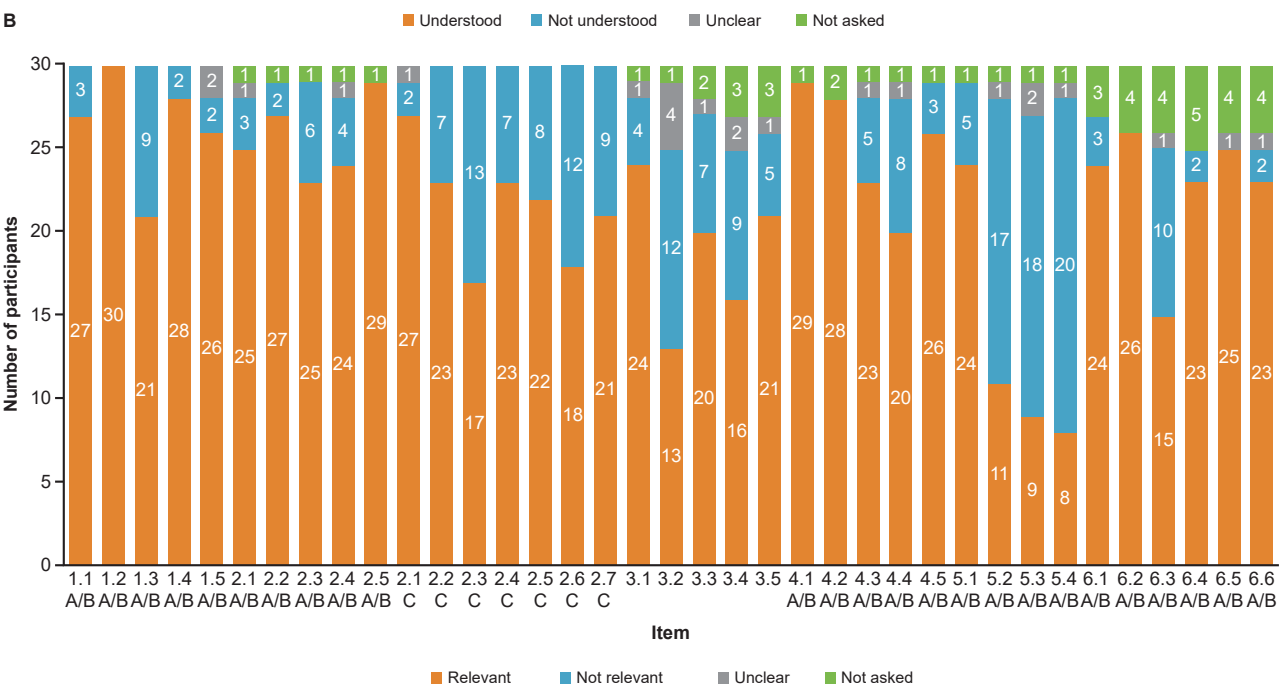

Supplement: Supplementary file 1 — Additional file 1 : Supplementary Materials. Supplementary Table S1. Description of RTDC qualitative tasks. Supplementary Table S2. Summary of impact concepts and sub-concepts reported in the CE interviews. Supplementary Table S3. Current treatment satisfaction and treatment goals, with example participant quotes. Supplementary Fig. S1. Symptom and impact level saturation analysis (total sample). Supplementary Fig. S2. An overview of the reported goals for treatment. Supplementary Fig. S3. Cognitive interview results showing (A) understanding and (B) relevance for MHQ items. [file 41687_2021_286_MOESM1_ESM.zip › GSK [2533] Suppl Figure 3_updated.pdf]

Number of participants

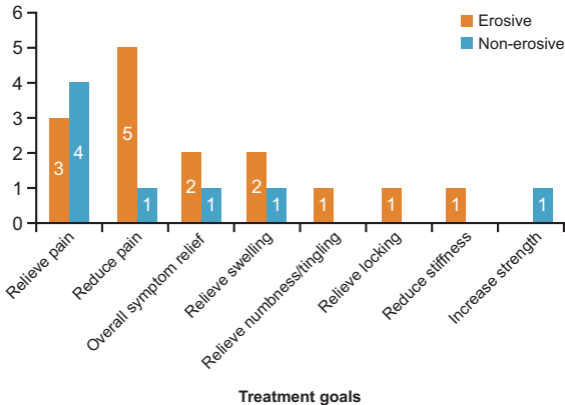

Supplement: Supplementary file 1 — Additional file 1 : Supplementary Materials. Supplementary Table S1. Description of RTDC qualitative tasks. Supplementary Table S2. Summary of impact concepts and sub-concepts reported in the CE interviews. Supplementary Table S3. Current treatment satisfaction and treatment goals, with example participant quotes. Supplementary Fig. S1. Symptom and impact level saturation analysis (total sample). Supplementary Fig. S2. An overview of the reported goals for treatment. Supplementary Fig. S3. Cognitive interview results showing (A) understanding and (B) relevance for MHQ items. [file 41687_2021_286_MOESM1_ESM.zip › GSK [8367] Suppl Figure 2.pdf]
